# Resolving Cattle GWAS Loci: Current Progress, Persistent Challenges and Future Directions

**DOI:** 10.3390/cimb48080852

**Published:** 2026-08-21

**Authors:** Aizhan Mussayeva, Lidiia Samarina

**Affiliations:** Institute of Genetics and Physiology, Almaty 050000, Kazakhstan

**Keywords:** cattle genomics, GWAS, fine-mapping, molecular QTL, regulatory variation, structural variants, pangenome

## Abstract

Genome-wide association studies (GWASs) have mapped many regions affecting cattle production, health and fertility, yet the lead variant is usually a marker for a linkage-disequilibrium block rather than the molecular lesion. This state-of-the-field review examines how those loci are interpreted at different levels. The literature shows both progress and persistent limits. Colocalization may identify a likely effector transcript without establishing mediation, whereas structural variants missing from SNP-based analyses can account for expression, splicing, or complex-trait signals. Earlier reviews have audited proposed causative variants across cattle and pigs, surveyed causal variants across livestock species, or concentrated on structural variation. Here, these lines of evidence are brought together around a narrower question: Why do cattle complex-trait loci remain resolved at such different biological depths? Coding, regulatory, splicing, and structural examples show where inference is persuasive and where alternatives remain. The evidence is organized as a conceptual landscape, not a validated hierarchy or prescriptive pipeline. The available mechanistic evidence is nevertheless concentrated in commercial taurine, particularly dairy populations, which limits the direct transferability of locus-level conclusions to indicine, African taurine, composite and locally adapted cattle. Priorities include clearer causal terminology, multi-signal and multi-breed analyses, better representation of structural variation, tissue- and cell-state-matched molecular data, native bovine experimental systems and transparent reporting of unresolved explanations.

## 1. Introduction

Sequence-level association has transformed cattle genetics. The first taurine reference genome, the Bovine HapMap and the 1000 Bull Genomes Project supplied a shared coordinate system, characterized breed differences in linkage disequilibrium (LD) and enabled sequence imputation at scale [1,2,3]. ARS-UCD1.2 then improved assembly contiguity and gene representation, although no linear reference captures all bovine sequence diversity [4]. Together, these resources made dense scans of production, fertility, health, efficiency and adaptation traits routine. However, the mechanistic interpretation has advanced more slowly. Recent selection, small effective population sizes and intensive use of influential sires have left long haplotypes in many cattle breeds. Within such regions, numerous variants can carry almost indistinguishable association statistics [2,5]. The lead SNP may be functional, but it may instead tag a coding allele, enhancer variant, splice-altering substitution, mobile-element insertion or structural variant. Because the LD phase also changes across breeds, an accurate predictor in one population may transfer poorly to another even when the underlying biological effect is real [5,6].

Whole-genome sequence imputation increases marker density without solving that ambiguity. Performance depends on the ancestry and haplotype coverage of the reference panel and deteriorates for rare alleles and poorly represented populations [7]. Evaluations in Nellore and Hanwoo cattle illustrate how panel composition changes genotype accuracy and therefore the apparent sharpness of an association peak [8,9]. Short reads create a separate, systematic gap: Complex insertions, duplications, repeats and rearrangements are harder to discover and genotype than SNPs [10,11,12,13,14,15,16,17,18,19,20]. Statistical fine-mapping replaces a single top marker with posterior probabilities or credible sets, making uncertainty more explicit [21,22,23]. Cattle studies have incorporated functional annotation, evolutionary constraint and pleiotropic effects, while multi-breed analyses use contrasting LD to reduce the candidate interval [6,24,25,26,27]. These methods can rank the variants supplied to them; they cannot recover an omitted allele or prove molecular function. Credible sets remain sensitive to genotype quality, LD estimation, the assumed number of causal signals and the priors used [21,22,23,24,25,26,28].

CattleGTEx connects inherited variation to expression and splicing across tissues [29]. Functional Annotation of Animal Genomes (FAANG) resources map promoters, enhancers, accessible chromatin and histone states, and single-cell atlases refine these annotations to particular cell populations [30,31,32,33,34,35]. Reproductive-tissue molecular quantitative trait locus (QTL) mapping, allele-specific expression (ASE) and experimental tests of splice-disrupting alleles have shown that genome-wide association study (GWAS) loci may act through transcript abundance, isoform choice or cis-regulatory imbalance [36,37,38,39]. Colocalization and Mendelian randomization (MR) provide complementary causal-inference tools for connecting molecular phenotypes to complex traits. Colocalization evaluates whether molecular and trait associations are compatible with a shared causal signal, whereas MR uses genetic variants as instrumental variables to test whether genetically predicted variation in a molecular exposure is consistent with a directional effect on the trait. Both approaches remain conditional on their model assumptions, local LD structure, instrument validity and adequate treatment of multiple causal signals [40,41,42,43]. Haplotype-resolved assemblies and graph genomes represent insertions, deletions, duplications, inversions and sequence absent from the Hereford-derived reference [11,12,13,14,15,16]. Structural variants are enriched among cattle molecular QTL, and recent studies have imputed them into large genotyped populations for trait association and fine-mapping [10,17,44]. A phased interval containing correlated small and structural alleles is therefore often a more realistic unit of analysis than an isolated SNP.

Few recent reviews are especially relevant and have analyzed the current state of the topic. In particular, Johnsson and Jungnickel audited the evidence for proposed causative variants in 13 cattle and pig genes, explicitly separating support for the associated locus, the causative gene, and the causative variant. Liu reviewed cattle structural variation with emphasis on long-read sequencing, pangenome graphs and near-complete assemblies. Balatsky and colleagues surveyed causal-variant classes, molecular consequences, identification approaches and breeding implications across mammalian and avian livestock [45,46,47].

The current review is cattle-specific and compares resolved and unresolved cattle loci, retains plausible alternative explanations and identifies areas in which the field lacks consensus. The objectives of the present review are to describe the current state of cattle GWAS locus resolution; evaluate the contributions and limitations of available statistical and functional approaches; compare representative examples of successful and unresolved interpretation; identify technical and biological bottlenecks; and outline priorities for research and resource development. We use ‘regulatory haplotype’ as a descriptive term for a phased segment carrying one or more candidate variants affecting gene expression, transcript processing or chromatin activity; the term does not imply cooperative function or a demonstrated mechanism [1,2,3,4,17,18,19,20,21,22,23,24,25,26,27,28,29,30,31,32,33,34]. An important limitation of the current evidence base is its population imbalance. Most deeply characterized loci, together with many of the largest sequence, imputation, molecular-QTL and pangenome resources, derive from commercial taurine populations, particularly dairy breeds. This matters because linkage disequilibrium (LD) phase and local haplotype structure differ among cattle populations, allele frequencies and potentially causal alleles may vary among breeds, and imputation accuracy declines when the reference panel poorly represents the target ancestry [5,6,7,8,9]. Structural and non-reference alleles are also incompletely represented by a single linear reference and may differ among populations [11,12,13,14,15,16,17]. Consequently, mechanistic conclusions obtained in Holstein, Jersey or other taurine populations should not be assumed to transfer unchanged to *Bos indicus*, African taurine, composite or locally adapted cattle. Cross-population comparisons should distinguish failure of marker transfer caused by differences in LD or allele frequency from genuine allelic or biological heterogeneity. This review presents an integrated assessment of locus-resolution bottlenecks—especially multiple causal alleles, breed-specific LD, tissue and cell-state specificity, structural-variant completeness and validation requirements.

## 2. Materials and Methods

### 2.1. Review Design and Scope

We conducted a focused narrative review of cattle GWAS locus interpretation. The design followed SANRA quality domains, including a stated question, a documented search and evidence-based reasoning; relevant PRISMA-S reporting elements were adapted for the search description [48,49]. This review was not registered as a systematic review, and no pooled effect estimate was planned. The central evidence base comprised cattle studies that connected a complex-trait association to a candidate gene, variant class or molecular mechanism. Priority was given to work combining two or more of the following: sequence association, conditional or Bayesian fine-mapping, multi-breed comparison, tissue-matched expression quantitative trait locus (eQTL) or splicing quantitative trait locus (sQTL), chromatin or cell-type annotation, ASE, a functional assay, long-read discovery, structural-variant genotyping or pangenome representation. Foundational genome, LD and imputation papers were included where they defined persistent limitations. Related reviews were retained to compare scope and conclusions.

### 2.2. Information Sources and Search Strategy

Targeted searches were performed through 27 July 2026 in PubMed and Crossref, supplemented by publisher websites and backward citation tracing. No starting year was imposed because historically resolved variants were needed as benchmarks; recent primary studies were emphasized for molecular QTL, single-cell resources, splicing assays and pangenome-based mapping. DOI metadata and publication status were checked against final publisher records [50,51]. Five concept groups combined “cattle OR bovine” with the following: (i) “GWAS OR genome-wide association” and “fine-mapping OR causal variant”; (ii) “eQTL OR sQTL OR molecular QTL” and “trait”; (iii) “pangenome OR structural variant OR long-read” and “association”; (iv) “allele-specific expression OR chromatin OR single-cell” and “regulatory variant”; or (v) “functional assay OR reporter assay” and “splicing OR regulatory”. A separate “review” search identified overlapping syntheses. Breed names, candidate genes and article titles were then used for locus follow-up and citation verification [30,31,32,33,34,35].

### 2.3. Eligibility Criteria and Study Selection

Peer-reviewed studies of *Bos taurus*, *Bos indicus* or admixed cattle were eligible if they reported a complex-trait association or a directly relevant molecular-QTL resource and allowed at least two links to be evaluated. These links included statistical localization, haplotype or cross-population comparison; tissue-specific molecular association; cell or chromatin localization; allele-level testing; structural-variant discovery; and phenotype-level validation. Resource papers were included when they defined an assembly, atlas, pangenome or shared infrastructure used by primary studies [36,37,38,39]. Studies that only listed significant markers, assessed genomic prediction without locus interpretation, described diversity without a mechanistic connection or annotated only the nearest gene were excluded from the central synthesis. Peer-reviewed versions replaced preprints where available, and non-cattle primary studies were excluded from the evidence tables. Reviews were used to map earlier coverage and terminology, not as primary support for individual loci. The criteria were applied during title-and-abstract screening and full-text assessment.

### 2.4. Data Extraction and Conceptual Synthesis

For each retained study, we recorded population and breed composition, sample size, phenotype, genome assembly, tested variant classes, genotype or imputation method, local and conditional association results, fine-mapping output, tissue or cell state, molecular phenotype, allele-specific or functional tests, effect direction, the authors’ causal statement and plausible alternatives. We also noted whether structural and non-reference alleles were represented and whether molecular evidence was phased to the trait-associated haplotype. Evidence was grouped by the question it addressed: Association links inherited variation to phenotype; fine-mapping localizes signals among tested variants; molecular QTL connect inherited variants to intermediate molecular phenotypes; colocalization evaluates whether trait and molecular associations are compatible with a shared causal signal; Mendelian randomization (MR) evaluates whether genetically predicted variation in a molecular exposure is consistent with a causal effect on the trait; chromatin and cell atlases define plausible biological context; ASE and functional assays test allele-dependent effects; long reads or pangenomes expand the variant catalogue. These approaches address different inferential questions and should not be treated as independent confirmations when they depend on the same local association signal or linkage-disequilibrium structure [52,53,54]. Four terms provide an interpretative vocabulary. A term ‘associated locus’ links a region to a phenotype; a term ‘mechanistic candidate’ has statistical and molecular support but retains alternatives; a term ‘functionally supported variant’ has an observed allele-dependent molecular effect; an ‘experimentally supported causal variant’ additionally connects that effect to the organism-level phenotype. The boundaries remain debated, and no study was assigned to a formal category by a scoring rule [50,51].

### 2.5. Synthesis and Methodological Limitations

Quantitative meta-analysis was inappropriate because phenotype definitions, breeds, variant catalogues, tissues and statistical outputs differed markedly. We therefore compared evidence types by the questions they addressed and selected representative cases spanning resolved coding variants, unresolved regulatory haplotypes, tissue-specific molecular QTL, tested splice variants and structural candidates recovered through pangenomes. Figures and Tables summarize this organization and the uncertainty retained in each case. This targeted narrative design cannot estimate the frequency of causal-variant classes, quantify publication bias or guarantee exhaustive retrieval. A global risk-of-bias score would also be misleading across such heterogeneous designs. Limitations were instead recorded for variant representation, population transferability, molecular context and validation, and conclusions were matched to the documented evidence rather than to the popularity of a technology or trait [48,49].

## 3. Why the Lead SNP Is Usually Not the Mechanism

### 3.1. Linkage Disequilibrium Defines the Candidate Space

LD defines what a cattle association study can resolve. Within a breed, several markers may track the same ancestral segment so closely that their likelihoods are nearly indistinguishable. Their ranking then reflects sampling noise, allele frequency, genotype quality and model specification, as well as proximity to the functional allele [5,21,24]. Reporting only the smallest *p* value hides this uncertainty and encourages a false transition from “associated variant” to “functional SNP.” Breed comparisons can shorten the candidate interval when a shared causal allele is carried on different haplotypes. Persistence of the LD phase is incomplete across Holstein–Friesian, Jersey and Angus cattle, and multi-breed mapping has improved resolution for some milk-production loci by exploiting that contrast [5,6]. The approach fails when breeds do not share the same causal allele, when the allele is rare or fixed in one population, or when phenotype definitions are not comparable. Lack of replication can therefore indicate different LD, allelic heterogeneity or different biology; it is not by itself a verdict on the original signal [5,6,26].

A favorable allele driven rapidly upward in frequency carries the surrounding haplotype, producing an extended region of correlated associations. In multibreed beef cattle, sweeps for increased stature impeded localization of variants affecting fertility and correlated traits [28]. Analyses of such regions should model multiple signals, examine local selection history and report sensitivity to the LD reference [21,22,23,24,25,28]. The relevant LD is the LD in the analyzed animals, not necessarily that of an external reference panel. Summary-statistic fine-mapping can become miscalibrated when the reference contains different breeds, generations or family structure, especially for low-frequency alleles. Within large half-sib families, recent recombination events can be more informative than population-wide correlations, but only if haplotypes are phased and transmission is modeled. Conversely, pooling breeds before defining local signals can average distinct causal architectures into one apparently broad locus. Study-specific LD matrices, family information and breed-stratified conditioning therefore provide complementary views of the same interval [21,22,23,24,25,26,28].

### 3.2. Variant Catalogues Are Unequal Across Populations

Dense imputation does not make all variants equally observable. Reference panels are strongest for breeds with many sequenced ancestors and recurrent haplotypes; accuracy falls for low-frequency alleles and genetically distant populations [7,8,9]. Because association evidence is conditional on genotype accuracy, candidate variants should be filtered or weighted using variant-level imputation metrics, not only an average accuracy for the dataset. Leave-one-breed-out or ancestry-matched evaluations are particularly informative when a result is intended to extend beyond a single commercial population [7,8,9].

Linear-reference mapping creates a second asymmetry. Reads carrying sequence absent from the reference can map poorly or not at all, and insertions are harder to represent than deletions relative to that reference. Haplotype-resolved assemblies have exposed tens of thousands of structural variants and non-reference sequences that conventional SNP arrays could not test [11,12]. The omission is systematic rather than random: Duplicated, repetitive and rapidly evolving regions are precisely those in which read mapping and short-read structural-variant genotyping are most difficult [11,12,13]. This asymmetry changes the meaning of an SNP credible set. If a nearby structural variant is untyped, a well-imputed proxy may receive high posterior probability: statistically useful, but mechanistically secondary. Pangenome-genotyped and long-read data have already identified structural variants as lead eQTL or sQTL and shown their enrichment among molecular QTL [10,17]. Candidate-set completeness must therefore be stated relative to the small and structural variants actually tested.

Completeness is also allele- and population-specific. One breed may carry a deletion, another a small regulatory substitution on the same haplotype background, and a third may carry neither allele. Collapsing these configurations into a biallelic marker can obscure allelic heterogeneity. Rare structural alleles pose a further problem: Even accurate long-read discovery may yield too few carriers for association, while short-read imputation can appear precise because common non-carrier haplotypes dominate the accuracy metric. Per-allele carrier counts, sequence validation and breed-specific concordance are therefore more informative than a single genome-wide imputation statistic [7,8,9,10,11,12,13,14,15,16,17,44].

### 3.3. Prediction, Localization and Causation Are Different Tasks

Prediction rewards out-of-sample accuracy, so a correlated marker can remain valuable while LD is stable. Fine-mapping aims to localize one or more responsible alleles; mechanistic studies ask how those alleles alter biology. The appropriate data and validation criteria therefore differ [5,21,22,23,24,25]. Deregressed breeding values combine information from relatives and a genetic-evaluation model, whereas direct measurements have a different error structure. Milk fat percentage, fat yield and milk volume are correlated but not interchangeable; clinical mastitis and somatic-cell traits overlap without measuring the same event. Apparent pleiotropy can therefore arise partly from shared records or mathematical coupling. Large sequence-based GWAS offer exceptional power, but mechanistic comparison still requires explicit phenotype definitions, measurement windows and molecular context [55,56,57,58].

A locus may consequently be well resolved for prediction and poorly resolved for biology. Within-population prediction depends on validation and calibration; causal interpretation depends on the local variant set, molecular phenotype and viable alternatives; cross-breed transfer depends on shared alleles and the LD phase [24,25,26,28,55,56,57,58]. Treating these endpoints separately prevents predictive success from being mistaken for molecular explanation. A rare allele can have a convincing molecular effect yet contribute little to prediction in a population where few animals carry it. Likewise, an expression change measured after disease onset may be biologically real but downstream of the trait. Study designs should specify whether the intended claim concerns marker utility, localization, molecular function or mediation. That statement determines which negative results are decisive: weak cross-breed prediction does not negate an allele-specific assay, and a reporter effect does not show that the allele explains the population association [36,37,38,39].

## 4. Contributions and Limitations of Current Evidence Types

### 4.1. Statistical Fine-Mapping and Cross-Population Resolution

Conditioning only on the lead SNP can leave secondary effects in the residuals, whereas fitting many nearly interchangeable markers can divide one effect across redundant variables. Bayesian methods such as FINEMAP and SuSiE express this ambiguity through posterior inclusion probabilities and credible sets [21,22,23]. Cattle applications have combined association data with functional enrichment, evolutionary conservation and pleiotropic effects [24,25]. Their output is a probability model over the supplied catalogue, not a declaration that the highest-ranked allele is causal.

Different algorithms can disagree without either being implemented incorrectly. Exhaustive or stochastic searches, variational approximations and animal-breeding models make different compromises between computation, signal number and prior structure [21,22,23,24,25]. Posterior probabilities may also be overconfident when the LD matrix is estimated with error or when summary statistics and LD come from different samples. For that reason, agreement across methods is most useful when their assumptions differ and the candidate survives sensitivity analyses; running several tools on the same incomplete catalogue does not create independent evidence [21,22,23].

Functional priors can improve separation when they are learned independently and matched to the phenotype. Coding sequence, conserved bases and active promoters or enhancers may deserve different prior weights, but a liver annotation is not equally informative for every trait. Reusing the focal association to define an annotation and then to prioritize the same locus creates circular support. Multi-trait models raise a related concern: they gain power from shared effects, yet correlated phenotypes or nearby alleles can imitate biological pleiotropy [21,22,23,24,25,26,28,30,31,32,33].

Cross-population mapping is most informative when the same allele is accurately genotyped for a harmonized phenotype in populations with different LD patterns. Meta-analyses across eight cattle breeds and, earlier, three breeds increased resolution for milk composition while revealing population heterogeneity [26,27]. Consistent direction across different surrounding haplotypes supports a shared candidate. Discordance is not automatically refutation; it directs attention to allele frequency, genotype quality, environmental context, phenotype scaling and allelic heterogeneity [5,6]. A fixed-effect meta-analysis seeks an average shared effect and gains power when directions agree; a heterogeneous or breed-stratified analysis asks whether the same model is plausible in every population. Trans-ethnic fine-mapping can narrow a shared interval only when the causal allele is present and the phenotype has comparable meaning. When those conditions fail, forcing one common effect may produce an attractive but artificial consensus peak. Breed-specific association plots, heterogeneity statistics and haplotype backgrounds should accompany the combined estimate [26,27].

A useful report gives the credible-set definition, local LD, named assembly, allele frequency by population, genotype quality, conditional signals and sensitivity to priors. Calibration can be explored through simulations based on observed LD, but follow-up in an independent population or molecular assay is more informative. These details allow a locus to be revisited when a new assembly or pangenome changes the candidate set [4,11,12,13,14,15,16,17,28].

Recent studies sharpen two practical aspects of fine-mapping. Wu et al. developed a genome-wide fine-mapping framework that incorporates global genetic architecture and functional information rather than restricting inference to isolated genome-wide significant loci [59]. Although its empirical applications were principally based on human complex traits, the framework is relevant to cattle because it makes explicit how genome-wide architecture and functional priors can influence local posterior probabilities; cattle-specific calibration remains necessary. In Nellore cattle, Mota et al. integrated reaction-norm GWAS with Bayesian fine-mapping to distinguish loci associated with genetic merit from those associated with environmental sensitivity of reproductive performance [60]. This design illustrates why context-dependent effects should not automatically be pooled with average genetic effects before localization. Both studies reinforce that posterior support is conditional on the phenotype model, variant catalogue, LD structure and prior assumptions rather than constituting proof of causality.

### 4.2. eQTL, sQTL and Colocalization

Molecular QTL map inherited variation to transcript abundance, splice-junction use or other intermediate phenotypes. CattleGTEx reprocessed 7180 RNA-sequencing samples across major tissues, providing a practical bridge from GWAS to gene regulation [29]. Its value is not the annotation of a nearby expressed gene. A tissue-matched eQTL or sQTL can implicate a distal effector and specify a molecular direction that can be compared with the trait effect [29,30]. Milk composition may be influenced by mammary epithelium, liver, adipose or endocrine tissues; fertility loci may act in testis, epididymis, vas deferens or the female reproductive tract. Molecular mapping in 118 bulls across three male reproductive tissues identified more than 41,000 regulatory loci and connected fertility QTL to tissue-relevant expression and splicing phenotypes [37]. An eQTL from an accessible but biologically remote tissue is not equivalent evidence.

Colocalization tests whether the association patterns for a trait and a molecular phenotype are compatible with a shared causal signal. This is more informative than overlaps between peak coordinates, yet it does not establish mediation. Two linked variants can imitate one shared signal, and the original coloc model assumes at most one causal variant per trait; later extensions allow multiple signals [40,41]. Conditional analyses and multi-signal fine-mapping should therefore precede colocalization, and the result should be interpreted alongside direction, tissue relevance and candidate-set completeness [24,29,37,52].

Matching the two datasets is as important as the statistical method. Allele frequencies and LD should be sufficiently similar, variants should use the same assembly and allele orientation, and the molecular cohort must contain enough carriers of the relevant haplotype. A colocalization failure can otherwise reflect ancestry mismatch, weak molecular-QTL power or omission of the functional variant rather than distinct biology. A strong result is also conditional on the priors assigned to trait association, molecular association and sharing. Reporting posterior support across plausible priors is more informative than a single thresholded label [29,37,40,41]. Total abundance can remain stable while exon inclusion, splice-site choice or transcript stability changes. CattleGTEx, reproductive-tissue mapping and reporter assays all show that sQTL can expose mechanisms missed by gene-level eQTL analysis [36,37]. Because both processes are tissue- and stage-dependent, evidence is strongest in the cell population and physiological state in which the trait develops [29,30,33].

Large RNA-sequencing compendia gain power by combining studies, breeds, ages and conditions, but residual batch effects and cell composition can attenuate or distort molecular QTL. Highly expressed genes and common junctions are also easier to map than transient or cell-restricted events. A null result is interpretable only with sample size, expression depth, genotype count and molecular-phenotype reliability. Conversely, a large eQTL is not automatically phenotypically important; a modest, well-timed effect in the correct cell type may be more relevant [29,30,33,37].

### 4.3. Chromatin Annotation and Cell-Type Resolution

FAANG was established to coordinate functional annotation across domesticated species; subsequent cattle datasets have mapped promoters, candidate enhancers, accessibility, histone states and CTCF binding [31,32,34,35]. A fine-mapped variant in an element active in the relevant tissue makes altered enhancer or transcription-factor activity plausible, but the evidence remains positional until the allele changes that element or its target-gene output. The nearest gene may not be the target, and one element can contact several promoters. Across-tissue correlations can also reflect composition or shared upstream regulation. In cattle, allele-specific histone or CTCF binding did not consistently predict ASE direction in correlated peak–exon pairs [39]. Chromatin evidence is therefore most useful when joined to eQTL, ASE, contact or perturbation data. The cattle multi-tissue atlas profiled about 1.79 million cells from 59 tissues and established reference cell identities and expression programs [33]. It can localize a candidate gene to a cell population, but an atlas based on relatively few animals is not a cell-type eQTL map. It defines context more readily than inherited regulatory effects [33]. A 2025 milk study combined GWAS data from more than 16,000 Holstein cattle with CattleGTEx cis-QTL data, the cattle cell atlas, and new mammary and liver RNA-seq and ATAC-seq data [61]. It nominated cell populations and regulatory candidates, although the small experimental cohort limited genotype-level inference. Developmental and environmental coverage is also thin: lactation stage, heat, diet, infection and age can remodel chromatin and cell composition. FarmGTEx identifies context-specific sampling as a next step; until those data mature, a missing annotation should not be read as evidence against regulation [30,31,32,33,61].

### 4.4. Splicing, ASE and Functional Assays

ASE compares parental alleles within the same animal, controlling many trans-acting and environmental effects. Consistent imbalance in heterozygotes supports a cis-regulatory difference, and tissue specificity helps localize it. An atlas of 7532 cattle RNA-sequencing datasets across 35 tissues found widespread, often tissue-specific ASE linked to promoters, enhancers, splicing and nonsense-mediated decay [38]. Each heterozygote provides an internal comparison, making ASE useful when conventional eQTL mapping is underpowered. Reference-bearing reads can map more readily, especially near indels and structural variants; phasing errors can reverse or dilute a haplotype effect. Copy number, imprinting and stochastic monoallelic expression can also mimic enhancer-driven imbalance. Variant-aware alignment, phased sites and replication across individuals are essential [38,39].

Splicing assays provide a route from prediction to direct molecular evidence. Charles and colleagues combined GWAS, deep-learning prediction and a massively parallel reporter assay; 38 associated splice-disrupting candidates yielded three variants described as causal by the authors [36]. The attrition matters. A high prediction score prioritizes an allele; a reproducible allele-dependent transcript demonstrates molecular function. Reporter assays remain reductionist: Minigene and massively parallel constructs can omit native chromatin, long-range contacts, developmental state and tissue-specific trans factors. A positive result shows that a sequence can alter expression or splicing under the assay conditions; a negative result may expose a poor model rather than an inert allele. The strongest regulatory cases combine a trait-associated haplotype, allele-dependent expression or splicing in the relevant tissue; concordant functional testing; and a direction consistent with phenotype. Endogenous editing, bovine primary cells, organoids or informative natural genotypes can strengthen this connection, but native bovine systems also have important practical limitations. Primary bovine mammary epithelial cells can rapidly lose tissue-specific function in culture: Expression of lactation-associated genes declines markedly after only three to four passages, although three-dimensional matrix culture can partially restore a more representative lactogenic phenotype [62]. Similar passage-dependent loss of physiological identity has been demonstrated in bovine luteal cells, in which repeated passaging reduced luteal marker expression, progesterone synthesis and proliferative capacity; short-term or very early-passage cultures retained molecular features most similar to the corpus luteum in vivo [63]. Thus, primary cells and three-dimensional cultures can improve biological relevance relative to simplified reporter systems, but their phenotype remains passage- and culture-dependent, and they are less scalable for systematic testing of many candidate alleles.

### 4.5. Structural Variation and Pangenome Representation

Structural variants can change dosage, disrupt exons, move regulatory elements, create sequence and reshape local genome structure. Their biological reach is matched by technical difficulty, a problem recognized in early livestock surveys and still evident in long-read studies [18,19,20]. Haplotype-resolved bovine assemblies recover tens of thousands of such alleles, including complex repeats and coding disruptions [11]. A global pangenome based on 898 animals from 57 breeds identified 83 Mb of sequence absent from the linear reference together with many deletions, inversions and duplications [12].

An assembly set emphasizes high-quality haplotypes; a variant graph genotypes known alternatives in many short-read samples; a breed-focused graph may improve local sensitivity while omitting diversity elsewhere. The Bovine Pangenome Consortium coordinates assembly production and access across global breeds [13]. Studies should state which animals and haplotypes enter the graph and how complex variants are projected to a common coordinate system [11,12,13]. Using 16 haplotype-resolved assemblies, Leonard, Mapel and Pausch found eQTL and sQTL for which a structural variant had the strongest association [10]. A separate global study of 2409 animals from 82 breeds found many structural variants to be poorly tagged by SNPs and identified an event with enhancer activity in an *IGFBP7*-positive cell context [14]. SNP-only candidate sets can therefore be biologically incomplete [10,14]. A French pangenome from 64 assemblies representing 14 dairy and beef breeds added 0.159 Gb beyond ARS-UCD1.2 and catalogued more than 100,000 structural variants [15]. A phased Holstein graph improved genotyping and linked structural alleles to economically important traits [16]. Portability still requires testing in indicine, admixed and locally adapted cattle [11,12,13,14,15,16].

Long-read cohorts provide a direct test without relying entirely on graph imputation. HiFi sequencing of 120 primarily Braunvieh bulls identified 79,300 structural variants and 27,300 testis molecular QTL; structural alleles were enriched among lead expression and splicing QTL [17]. Imperfect structural-variant genotyping nevertheless reduced discovery, showing that long reads improve rather than eliminate technical uncertainty [17,19].

A July 2026 study imputed more than 68,000 structural variants into 50,299 dairy bulls, tested 43 traits and fine-mapped 32 high-confidence structural candidates [44]. The study joins assembly discovery, graph genotyping, imputation and GWAS, but the remaining work is locus-specific: Which allele changes molecular function, and does that mechanism generalize beyond the analyzed population? Structural variants should not be privileged because they are large, nor should SNPs be privileged because they are convenient. Both belong in the same phased comparison, with genotype quality, LD, molecular evidence and segregation evaluated together [10,11,12,13,14,15,16,17,44].

A WCGALP 2026 report from French dairy cattle illustrates the same issue at population scale. Naji et al. constructed a pangenome-informed structural variant (SV) reference; genotyped long-read-discovered alleles in short-read samples; imputed them into Holstein, Montbéliarde and Normande bulls; and jointly tested SNVs and SVs for production, health, fertility and stature traits [64]. Thirty-six unique SV–trait associations were reported, and conditional analysis prioritized 10 SVs within QTL for milk composition and stature. The very low concordance observed for duplications relative to deletions and insertions is equally informative: Expanding the catalogue without variant-class-specific benchmarking can replace omission with genotype errors. These results support joint small-variant/SV analysis but not automatic causal assignment to a structural allele.

Representation itself can change the apparent allele count. Callers may describe one rearrangement as a single insertion, several nearby breakpoints or a copy-number state; graph paths can also encode nested alleles that have no one-to-one linear coordinate. Before association, structural calls need harmonization by sequence, breakpoint and haplotype rather than proximity alone. After association, the reported object should include the inserted sequence or graph path, copy number, breakpoint confidence and orthogonal validation. Without those details, another study may test a related but non-equivalent allele under the same locus label.

### 4.6. Mendelian Randomization, Direction, Mediation and Pleiotropy

Mendelian randomization (MR) provides an additional causal-inference layer between molecular association and experimental validation. In this setting, inherited genetic variants are used as instrumental variables for an exposure, such as gene expression, transcript abundance or another molecular phenotype, to test whether genetically predicted differences in that exposure are consistent with a causal effect on a complex trait [42,43]. Valid interpretation requires three central instrumental-variable assumptions: The genetic instrument must be associated with the proposed exposure (relevance); it should not be associated with factors that confound the exposure–outcome relationship (independence); it should influence the outcome through the proposed exposure rather than through an alternative pathway (exclusion restriction) [43]. These assumptions are particularly important for molecular MR within cattle GWAS loci because long-range linkage disequilibrium (LD), multiple cis-regulatory signals and pleiotropic variants can make apparently strong instruments difficult to interpret. Weak molecular QTL, population structure, inaccurate imputation and overlap between molecular and trait datasets can introduce additional uncertainty.

MR addresses a different question from statistical fine-mapping and colocalization. Fine-mapping asks which variant or set of variants is most compatible with the observed trait association among the alleles tested. Colocalization asks whether association patterns for a molecular phenotype and a complex trait are compatible with a shared causal signal. MR instead asks whether the genetically predicted molecular exposure is consistent with a directional causal effect on the phenotype [21,22,23,24,25,40,41,42,43]. These distinctions matter because a colocalized eQTL and GWAS signal does not establish that altered expression mediates the trait effect, whereas an MR association can also arise when the instrumental variant influences the outcome through horizontal pleiotropy or when linked variants separately influence the exposure and trait. Consequently, agreement among fine-mapping, colocalization and MR strengthens prioritization of a mechanistic hypothesis but does not constitute three independent demonstrations of causality when the analyses depend on the same local variants and LD structure [52,53,54].

Recent cattle studies illustrate both the value and the limitations of this integration. Teng et al. combined GWAS, Bayesian fine-mapping, cis-eQTL-based MR and colocalization to prioritize genes affecting milk-related traits in dairy cattle [52]. Other integrative studies have likewise used molecular-QTL information and genetically predicted expression to prioritize candidate genes for complex cattle traits [53,54]. Such analyses can substantially narrow the transition from an associated locus to a plausible effector gene, especially when the direction of the molecular effect is biologically coherent and supported in a relevant tissue. However, MR evidence should be interpreted together with instrument strength, local LD, the possibility of multiple causal variants, tissue relevance and sensitivity to pleiotropy rather than as proof that the molecular phenotype mediates the organism-level trait. Where possible, alternative instruments, conditional molecular-QTL signals, colocalization after multi-signal fine-mapping and independent molecular datasets provide useful sensitivity tests [37,40,41,42,43,52,53,54].

Direction and mediation remain distinct even when multiple statistical layers agree. An allele that raises both transcript abundance and a trait does not demonstrate that expression mediates the phenotypic effect; the molecular and phenotypic changes may instead be parallel consequences of the same variant or of correlated variants. Direction is more credible when the implicated gene has a coherent role in the relevant tissue, the molecular change occurs in the physiological state in which the phenotype develops, and perturbation reproduces the predicted downstream effect [31,32,33,52,61]. In endocrine and metabolic networks, expression may also respond to physiology rather than initiate it. Tissue-specific molecular QTL, trait-specific conditioning and recombinant haplotypes can help discriminate among these alternatives [25,28,37].

Pleiotropy itself can reflect several architectures: One allele may alter one biological process that affects several traits, one allele may independently influence several molecular phenotypes, or linked alleles may act on different traits. Multi-trait association or MR alone cannot unambiguously distinguish these models [42,43]. This distinction matters for breeding and genome editing because a truly pleiotropic allele may impose correlated consequences, whereas effects generated by linked variants may potentially be separated. Regulatory effects may also be restricted to lactation, infection, heat stress, development or other physiological states, and averaging across states can attenuate or alter the apparent molecular effect [30,33]. Mediation is therefore most persuasive when genotype precedes a molecular change in the relevant tissue and state, statistical evidence is compatible with a directional exposure–outcome relationship, perturbation produces the expected downstream biology, and plausible alternative explanations fit the data less well. Complete experimental proof may be impractical in cattle, but convergence from MR, colocalization, natural recombinants, family segregation, repeated tissue cohorts and orthogonal functional assays can progressively strengthen causal inference. Causal terminology should nevertheless stop at the strongest link directly supported by the available evidence [42,43,50,51,52,53,54,61,65]. 

## 5. Conceptual Landscape of Evidence Used in Locus Interpretation

### 5.1. Complementary Evidence Types

**Figure 1:**
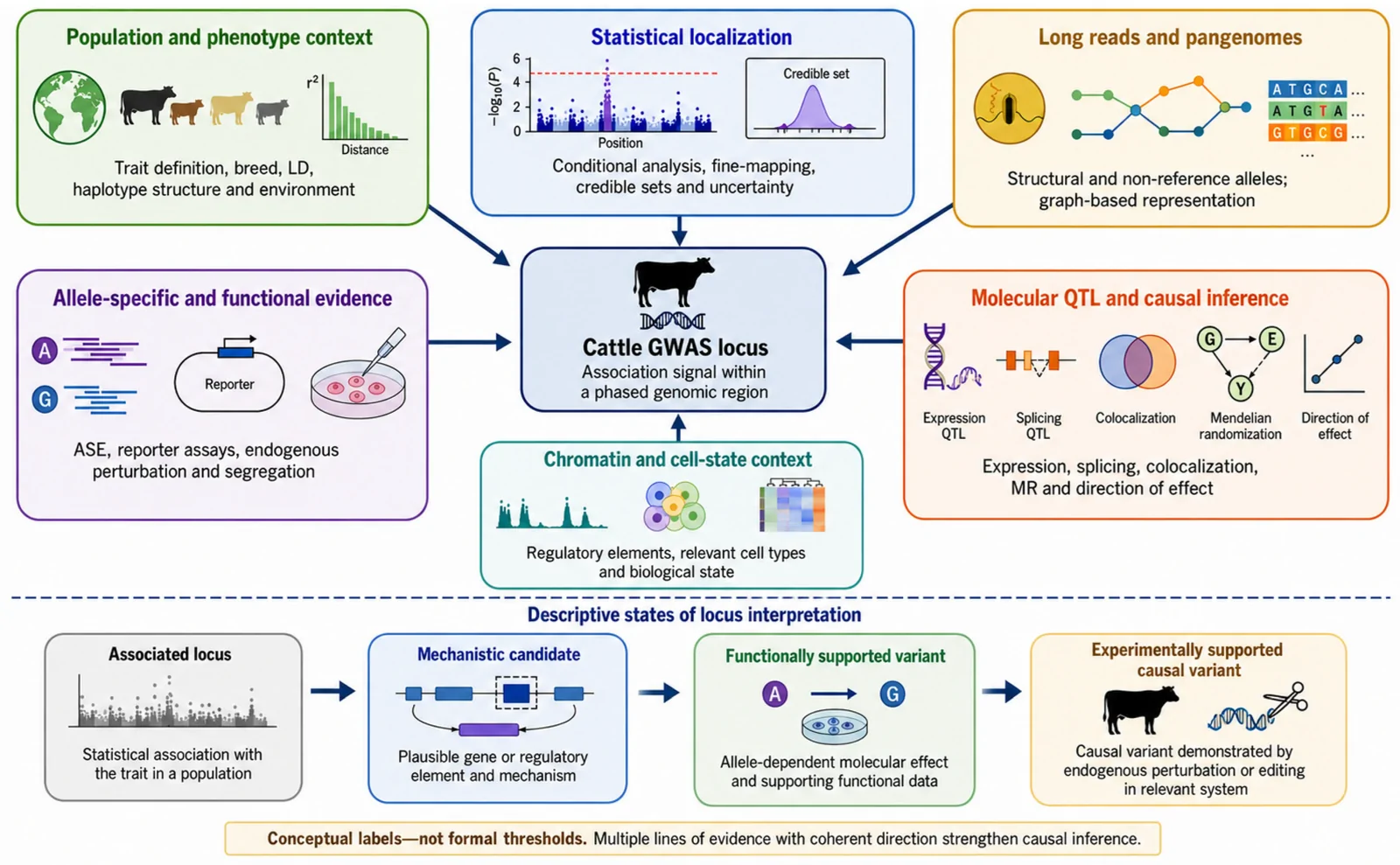
Conceptual landscape of complementary evidence used to interpret cattle GWAS loci. Population and phenotype information defines the biological and genetic context of an association signal; statistical fine-mapping localizes candidate variants among the alleles tested; molecular QTL connect genetic variation to expression or splicing; colocalization evaluates whether molecular and trait associations are compatible with a shared causal signal; Mendelian randomization (MR) evaluates whether genetically predicted variation in a molecular exposure is consistent with a directional effect on the trait. Chromatin and cell-state data identify plausible regulatory contexts, allele-specific and functional assays test molecular consequences, and long-read sequencing and pangenomes expand the candidate set to structural and non-reference alleles. These evidence types are complementary but address different inferential questions, and several may depend on the same local linkage disequilibrium structure. Their arrangement therefore does not represent a required analytical sequence, evidence hierarchy or decision rule. The descriptive states shown below the landscape indicate possible levels of locus interpretation rather than formally validated classes or thresholds.

Current cattle studies use several complementary forms of evidence to move from an associated locus toward a molecular explanation. Population association and haplotype information define the statistical signal and its breed-specific context, whereas conditional analysis and fine-mapping determine which variants remain plausible within the tested catalogue. Molecular QTL connect inherited variation to intermediate phenotypes such as gene expression or splicing. Colocalization then asks whether the molecular and complex-trait associations are compatible with a shared causal signal, while Mendelian randomization (MR) asks whether genetically predicted variation in the molecular phenotype is consistent with a directional effect on the trait [52,53,54]. Chromatin and single-cell data help identify the relevant regulatory element, tissue or cell state; allele-specific expression and functional assays test allele-dependent molecular effects; long-read sequencing or pangenome analysis can reveal structural and non-reference alleles that were absent from the original candidate set [31,32,33,34,35,36,37,38,39,44]. These approaches answer different questions and should therefore be interpreted as complementary rather than as successive steps in a fixed validation pipeline. Figure 1 summarizes the conceptual landscape around the cattle GWAS locus.

Table 1 summarizes the contribution and principal limitations of each evidence type shown in Figure 1. The framework should not be interpreted as an evidence ladder. For example, colocalization can provide strong evidence that two association signals are shared without demonstrating molecular mediation, and MR can support a directional exposure–outcome hypothesis while remaining sensitive to pleiotropy and LD. Similarly, a functional assay may establish an allele-dependent molecular effect without showing that this effect explains the population-level trait association. The interpretation of each result therefore depends on the local genetic architecture, phasing, tissue and cell-state relevance, completeness of the variant catalogue and independence of the supporting evidence [21,22,23,24,25].

### 5.2. Areas Without Field-Wide Consensus

Causal vocabulary remains inconsistent. “Candidate gene,” “functional variant,” “quantitative trait nucleotide” and “causal variant” are applied to evidence ranging from proximity to phenotype-level validation. The terms used here preserve those differences but do not constitute a universal classification. The unresolved issue is how molecular effects, segregation, endogenous perturbation and population replication should constrain causal wording [61,65]. eQTL and sQTL may implicate different transcripts; a chromatin peak may map to several genes; ASE may appear only in a subset of tissues. A single score can conceal conflict and dependence, especially when analyses reuse animals or local LD. Multiple causal alleles make the problem harder because fine-mapping and colocalization tools differ in their assumptions about signal number. Transparent presentation of concordant and discordant layers is more defensible than an assumed hierarchy [37,38,39,40,41,52,61].

Population and variant representation create a second cluster of open questions. Cross-breed analysis can narrow a shared allele, but failed replication may reflect frequency, imputation, phenotype or genuine allelic heterogeneity [26,27]. Pangenome graphs reveal variants absent from SNP pipelines, yet graph construction, normalization and imputation remain method-dependent. Small and structural alleles should be compared within the same phased locus whenever possible, without privileging either class [44].

Finally, tissue relevance and functional validation cannot be reduced to one required package. A regulatory effect may be confined to lactation, infection, heat stress, development or a rare cell population, making a negative result in unmatched bulk tissue weak evidence. Reporter assays test sequence activity but omit native context; endogenous perturbation is more physiological but harder in cattle; segregation and natural recombinants provide in vivo evidence but are uncommon. The field needs claim-specific standards that recognize these trade-offs rather than a single universal threshold [61,65].

### 5.3. Reporting, Reanalysis and a Cattle Locus Dossier Standard

Reanalysis of cattle GWAS loci requires preservation of the information needed to reconstruct both the statistical signal and its biological interpretation. We therefore propose a Cattle Locus Dossier Standard as a minimum reporting framework for locus-level studies (Table 2). The dossier is intended as a reporting standard rather than a causal score or decision algorithm. Its purpose is to make the assumptions, tested variant space and remaining uncertainty at each locus explicit and reusable.

At minimum, a locus dossier should identify the phenotype and study population, genome assembly and coordinate system, tested variant classes, allele definitions, genotype or imputation quality, cohort-specific local linkage disequilibrium (LD), conditional signals or credible sets, and haplotype/phasing information. For molecular-QTL evidence, the relevant tissue or cell state, molecular phenotype and phase relationship between the molecular-QTL allele and the trait-associated haplotype should be reported. Complex structural variants require additional sequence-level description because a linear coordinate alone may not uniquely identify the allele; breakpoint coordinates, inserted sequence or copy-number state, graph path where applicable, and orthogonal validation should therefore be provided [37,44].

The dossier should also record competing candidate variants or genes, negative results and unresolved explanations. A high-probability SNP from an earlier fine-mapping study may remain an effective predictor while later becoming a mechanistically secondary proxy after discovery of an untyped structural or regulatory allele on the same haplotype. Preserving the original candidate set, LD structure, analytical assumptions and uncertainty allow such reinterpretation without treating scientific revision as contradiction [21,22,23,24,25,26,44].

## 6. Representative Resolved and Unresolved Loci

Large-effect coding alleles provide benchmarks for relatively advanced interpretation. At *DGAT1*, dense mapping and association converged with biochemical evidence that K232A changes enzyme activity and with the direction of milk-composition effects [50]. The *ABCG2* Y581S allele was supported by fine localization and transporter biology [51]. At *GHR*, an F279Y substitution emerged from QTL dissection and was associated with major milk effects, while a deletion in *MSTN* explained double muscling in Belgian Blue cattle [66,67]. These loci are unusually tractable because the protein change is interpretable and the phenotype is large; they are not templates for every complex-trait signal.

This contrast is important because many economically relevant cattle traits have a highly polygenic architecture in which numerous loci of small effect contribute to genetic variation. In Holstein cattle, for example, milk-fat percentage combines a major-effect locus with many loci of very small effect, whereas overall type was consistent with an architecture dominated by many small-effect loci [68]. Genome-wide analyses across multiple dairy traits likewise indicate widespread QTL effects and emphasize that individual causal effects are often small and difficult to separate from correlated variants in linkage disequilibrium [25]. This architecture is conceptually compatible with the omnigenic model proposed by Boyle et al. [69], which distinguishes “core genes” with relatively direct effects on trait biology from a much larger set of “peripheral genes” for which their effects may be transmitted through interconnected regulatory networks. The omnigenic model was developed primarily from human complex-trait genetics and should therefore be regarded as a conceptual framework rather than an established cattle-specific model. Nevertheless, it provides a useful perspective for cattle GWAS interpretation: Large-effect coding loci such as *DGAT1* or *ABCG2* may sometimes be resolved to a single quantitative trait nucleotide, whereas small-effect loci may reflect multiple regulatory variants, allelic heterogeneity, or perturbation of broader molecular networks. For these loci, resolution of the effector gene, regulatory haplotype, relevant tissue or cell state, and affected molecular pathway may represent a biologically informative intermediate endpoint even when the contribution of individual nucleotide variants cannot yet be separated. This does not reduce the value of variant-level fine-mapping; rather, it requires that the strength and granularity of the causal claim match the genetic architecture and the evidence available at each locus [61,65,68,69]. At the bovine stature locus, Karim and colleagues localized variants that modulate a chromosome domain containing *PLAG1*; later functional work strengthened *PLAG1* as the effector while leaving the precise contribution of correlated regulatory alleles open [70,71]. Reciprocal mutations in *PRL* and *PRLR* were mapped through informative phenotypes, segregation and physiological observations in hairy and slick cattle [72]. Such cases benefit from distinctive phenotypes or pedigrees that are rarely available for small polygenic effects.

An emerging example concerns *L-LCORL*. Majeres et al. reported functional and mechanistic validation of truncating *L-LCORL* variants associated with increased growth across domesticated species [73]. Cross-species recurrence and experimental functional evidence strengthen the biological plausibility of the gene, while the cattle claim still requires explicit separation of the focal truncation from correlated selection-haplotype variants and evaluation of pleiotropic effects. This example fits between an associated growth locus and a fully resolved cattle quantitative trait nucleotide: it advances allele-level function but does not make breed portability or organism-level trade-offs automatic.

The *MGST1* milk-fat locus illustrates a regulatory endpoint with residual variant uncertainty. Sequence-based association in 64,244 taurine cattle identified a cluster of 17 non-coding variants spanning *MGST1*, while mammary RNA sequencing in 375 lactating cows revealed a strong *MGST1* eQTL aligned with milk-composition effects [65]. The study makes *MGST1* a persuasive effector gene and supports a regulatory mechanism, but correlated non-coding candidates prevent assignment of the effect to a single nucleotide. Calling the locus a regulatory haplotype is more accurate than naming the lead SNP as causal [65].

Male fertility shows why tissue-matched molecular phenotypes matter. Mapping in testis, epididymis and vas deferens connected fertility QTL to expression and splicing effects, including a candidate molecular phenotype involving *WDR19* [37]. The study did more than show that genes near a fertility GWAS peak are expressed in reproductive tissue: It tested hundreds of thousands of molecular phenotypes against sequence variants in the relevant organs. At the same time, colocalization and molecular association nominate mechanisms; they do not replace allele-level perturbation [37].

The splice-disrupting variant study provides a useful validation funnel. GWAS and prediction algorithms generated candidates, but massively parallel reporter testing reduced the set to experimentally supported effects and identified three variants considered causal in the study. The contrast between 38 associated splice-disrupting variants and the smaller causal subset is scientifically important. It quantifies how much uncertainty remains after association plus in silico annotation and shows why a review should report attrition rather than only successful examples [36].

Recent milk-trait studies demonstrate both the value of integrating complementary causal-inference methods and the danger of treating concordant statistical results as independent evidence. Teng et al. combined GWAS, Bayesian fine-mapping, Mendelian randomization (MR) and colocalization to prioritize genes underlying milk-related traits [52]. These analyses address distinct questions: Fine-mapping prioritizes candidate variants, colocalization evaluates whether molecular and trait associations are compatible with a shared causal signal, and MR evaluates whether genetically predicted variation in the molecular exposure is consistent with a directional effect on the trait. Other studies have combined GWAS with cis-QTL, single-cell data, ATAC-seq, RNA-seq, genetically predicted expression or physiological annotation [53,54,61]. Earlier DNA/RNA sequence integration at the lactose locus likewise highlighted transport genes, and cross-breed sequence meta-analysis refined milk-composition QTL [27,74]. Convergence across these layers strengthens a mechanistic hypothesis, but analyses driven by the same local variants and LD structure are not independent replications. Direct allele-level or endogenous functional testing therefore remains important before statistical evidence is interpreted as molecular mediation [52,53,54,61]. Large sequence-based GWAS illustrate the current gap between discovery scale and mechanistic resolution. Analyses of approximately 180,000 German Holstein cattle and more than 50,000 Holstein bulls have produced many loci and fine-mapped candidates across milk, health and conformation traits [55,56,57]. Their scale yields precise prediction and association estimates, but long LD and imputed genotypes still leave mechanistic ambiguity. These datasets are powerful discovery resources and become more informative for mechanisms when paired with tissue-matched molecular QTL and more complete small- and structural-variant catalogues [36,37,38,39,44]. Meta-analysis across six dairy breeds increased power and identified biologically relevant candidate regions, but clinical mastitis and somatic-cell phenotypes do not have identical biological meanings [58]. Shared association across breeds can narrow a locus only when trait definitions and causal alleles are comparable. The case illustrates why phenotype harmonization and the distinction between shared biology and shared marker prediction remain central to cross-breed interpretation [5,6,26,58]. Structural variants have emerged as lead molecular QTL, breed-pangenome candidates and large-cohort trait associations, including 32 fine-mapped structural candidates in more than 50,000 dairy bulls [10]. These results do not imply that structural variants explain every unresolved locus. They demonstrate that variant representation was a limiting step and support future comparisons of structural and small variants within the same statistical and functional context [10,11,12,13,14,15,16,17,44].

**Figure 2:**
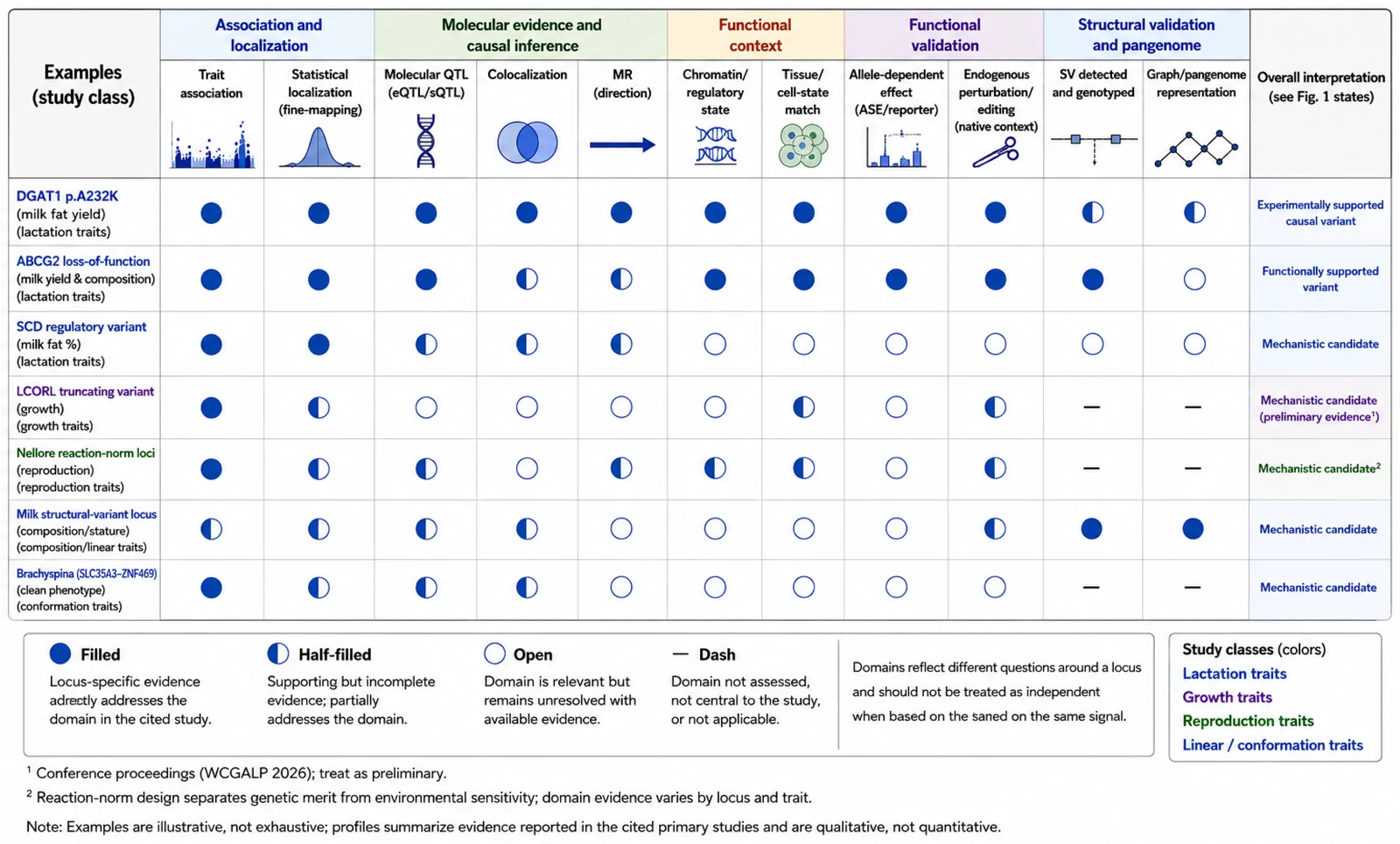
Illustrative evidence profiles and residual uncertainty across representative cattle loci and study classes. Filled symbols indicate locus-specific evidence directly addressing the corresponding domain; half-filled symbols indicate supporting but incomplete evidence; open symbols indicate a relevant domain that remains unresolved; dashes indicate evidence that was not central, not assessed or not applicable. Assignments summarize the evidence reported in the cited primary studies and are qualitative rather than quantitative. The matrix is not an additive score, validated hierarchy or decision rule.

Figure 2 applies the qualitative assignment to illustrate how representative cattle loci differ in the types of evidence available and in the uncertainties that remain. The profiles are intended to make differences among evidence domains explicit, not to rank loci using an aggregate score or threshold. The coding examples contain stronger allele-level evidence; *MGST1* is better resolved at the effector-gene level; reproductive molecular QTL emphasize tissue context; reporter-tested splice variants demonstrate molecular function; pangenome studies recover structural candidates that still need locus-specific interpretation [36,37,50,51,65].

The examples differ because their residual uncertainties differ, not because one technology always outranks another (Table 3). They also show that sample size and mechanistic depth are partly orthogonal. Hundreds of thousands of animals can estimate a small association precisely while leaving the allele unresolved; a focused molecular cohort may demonstrate an allele-dependent transcript but estimate population effects poorly [55,56,57]. The strongest designs connect a large discovery population, a deeply phenotyped molecular subset and an independent validation cohort carrying informative alleles or recombinant haplotypes. Across milk, fertility, mastitis and conformation, the same bottlenecks recur: LD, incomplete variant representation, tissue choice and validation [58,61,65]. Comparing how those bottlenecks were handled is more informative than cataloguing every significant marker.

## 7. Current Bottlenecks and Future Perspectives

The field would benefit from consensus statements that define the claim being standardized. Standards for an “associated locus” need not match those for a “causal variant,” and requirements for a coding allele with a large effect need not match those for a state-specific enhancer. At minimum, reports should distinguish locus localization, effector-gene assignment, allele-dependent molecular function and organism-level causality. Consensus is also needed on multiple causal alleles, integration of dependent molecular-QTL layers and the treatment of evidence that is strong in one breed but absent in another. These questions are scientific, not merely editorial, because the chosen term shapes downstream validation and breeding decisions [50,51,52,61,65].

These standards should also recognize differences in genetic architecture: The appropriate endpoint for a large-effect coding locus may be nucleotide-level resolution, whereas, for highly polygenic regulatory loci, the strongest defensible interpretation may initially concern an effector gene, regulatory haplotype or molecular network rather than a uniquely resolved nucleotide [25,68,69]. The next advance will not come from a larger catalogue of GWAS hits alone. CattleGTEx achieved scale by reusing public data, whereas reproductive and trait-focused studies gained biological specificity in smaller cohorts [29,37,61]. Future designs should match genotype, direct phenotype and molecular measurement in the state in which the trait develops. Longitudinal or minimally invasive sampling during lactation, heat stress, infection, development and reproduction would make genotype-by-state effects testable rather than speculative [30,33,37,61].

Such cohorts should be planned around molecular-QTL power, not only phenotype collection. Carrier count, tissue heterogeneity, sequencing depth, repeated measures and the expected frequency of the relevant cell population all determine whether a cis effect is detectable. Nested designs are realistic: a large genotyped discovery population, a smaller tissue cohort selected by haplotype and phenotype, and a still smaller experimental set for endogenous testing. Deliberate sampling of recombinants and rare structural-variant carriers can yield more locus information than a random molecular subset of the same size [37,38,44].

Population breadth must increase at the same time. Commercial taurine breeds, particularly dairy, dominate sequence, imputation, molecular-QTL and pangenome resources, whereas *Bos indicus*, African taurine, composite and locally adapted cattle remain less densely represented. This imbalance has several consequences for locus interpretation. First, differences in LD decay and persistence of phase alter both fine-mapping resolution and the portability of associated marker alleles across populations [5,6]. Second, allele frequencies can differ substantially among breeds, and the same genomic region may contain different causal alleles or haplotypes, so failure of replication does not by itself distinguish poor marker transfer from genuine allelic heterogeneity [26,27,28]. Third, sequence imputation and downstream association are less reliable when reference panels provide inadequate ancestry and haplotype coverage, as illustrated by breed-specific evaluations in Nellore and Hanwoo cattle [7,8,9]. Fourth, structural and non-reference sequence diversity is not uniformly represented across populations; haplotype-resolved assemblies and cattle pangenomes show that a candidate set defined in one ancestry may omit alleles occurring in another [11,12,13,14,15,16,17]. Haplotype-aware sampling should therefore connect diverse assemblies to phenotyped descendants, use ancestry-matched imputation panels where possible and avoid concentrating only on related elite sires. Cross-population analyses should report breed-specific allele frequencies, local LD, genotype quality and conditional association patterns before interpreting transferability. Greater representation of indicine, African, composite and locally adapted populations would improve both discovery and the recombination-based separation of candidates that remain inseparable within long taurine haplotypes [5,6,7,8,9,11,12,13,14,15,16,17]. Pangenome methods need explicit genotype benchmarking. Graph construction, structural-variant normalization and imputation may represent the same complex allele differently. Trio consistency, orthogonal long-read validation, graph-to-linear coordinate mapping and agreement across callers should accompany association results. Large-scale structural-variant imputation demonstrates feasibility, but long-read molecular-QTL work shows that genotype errors still cost power [44].

Mechanistic testing needs more native bovine systems, but their biological and technical constraints should be considered explicitly. Reporter assays remain scalable, whereas primary bovine cells and three-dimensional cultures provide a more native cellular context but are limited by passage-dependent phenotypic drift and dependence on culture conditions [62,63]. Embryo editing introduces additional constraints. In bovine zygotes, precise knock-in efficiency is strongly dependent on the repair strategy: A direct comparison reported 13.8% knock-in using a conventional homologous-recombination donor compared with 37.0% using a homology-mediated end-joining donor [75]. Mosaicism is also protocol-dependent; one bovine study observed 100% mosaicism among edited embryos following conventional microinjection at 20 h post-insemination, whereas earlier delivery of CRISPR components reduced this proportion to approximately 10–30% [76]. Generation of live validation animals additionally requires embryo production and transfer, pregnancy, genotyping and subsequent phenotyping, and the long generation interval of cattle limits experimental throughput even when editing is technically successful [77]. CRISPR perturbation in primary cells, organoids or embryos should therefore be used where the expected gain in causal resolution justifies these constraints. Candidate selection should begin from a phased locus containing correlated small and structural alleles so that the most convenient proxy is not tested by default [62,63,75,76,77]. Discovery and confirmation also need clearer separation. Reusing one GWAS to choose a locus, learn a prior, test colocalization and construct a pathway can create several concordant-looking results from one statistical signal. Independent tissue cohorts, orthogonal assays and external phenotype data are more informative than another annotation from the same animals. Environmental robustness should be tested as well: An effect observed in a high-input dairy system may differ under heat, nutritional restriction or pathogen exposure. Shared samples, shared LD and the environmental domain of the claim should be stated explicitly [21,22,23,24,25,26,52,61].

Finally, uncertainty must be reported in a reusable form. We propose that locus-level studies follow the Cattle Locus Dossier Standard summarized in Table 3, including explicit genome coordinates and assembly, allele definitions, tested variant classes, genotype quality, credible sets, cohort-specific LD, haplotype phase, molecular-QTL phase relative to the focal trait-associated haplotype, tissue metadata, competing candidates, and negative results. For structural variants, sequence- or graph-level allele definitions and orthogonal validation are particularly important. Standardized reporting would allow loci to be re-evaluated as assemblies, pangenomes, molecular atlases and functional data improve, without requiring previous uncertainty to be reconstructed retrospectively [37,38,39,44].

## 8. Conclusions

Technical progress has made cattle GWAS loci easier to interrogate but not uniformly easier to explain. Fine-mapping narrows the statistical alternatives; molecular QTL connect loci to expression or splicing; chromatin and single-cell data identify plausible contexts; functional assays test allele-dependent effects; long reads or pangenomes recover candidates missing from a linear reference. None of these observations alone establishes the full path from genotype to organism-level phenotype. Breed-diverse pangenomes, matched genotype–phenotype–molecular cohorts, longitudinal and cell-resolved sampling, native bovine perturbation systems, formal method benchmarking and publication of negative evidence are the most immediate priorities. They can move more loci from statistical association toward experimentally supported mechanisms without assuming that one pipeline will fit every trait, breed or variant.

## Figures and Tables

**Table 1:** Complementary evidence types currently used to interpret cattle GWAS loci.

Evidence Type	Contribution to Interpretation	Common Limitation
GWAS and haplotype association	Identifies a genomic interval that predicts the phenotype in the analyzed population [5,6,7,24,25,26,28].	Usually does not identify the causal allele, effector gene or molecular mechanism.
Conditional and Bayesian fine-mapping	Prioritizes one or more variants among those tested and can separate multiple local signals [21,22,23,24,25,26,28].	Results depend on LD, priors, genotype quality and completeness of the variant catalogue.
Tissue-matched eQTL/sQTL and colocalization	Links trait association patterns to expression or splicing phenotypes in a relevant tissue [29,37,40,41,52].	A shared signal does not by itself establish mediation, direction or a single causal variant.
Chromatin and cell-type annotation	Identifies plausible regulatory elements, tissues and cell populations [31,32,33,61].	Element activity does not establish the target gene or an allele-dependent effect.
ASE and molecular functional assays	Demonstrates a cis-regulatory, transcript-processing or other allele-dependent molecular effect [36,38,39].	The measured molecular effect may not explain the organism-level phenotype.
Long reads and pangenome genotyping	Expands the candidate set to structural and non-reference alleles missed by SNP-only analyses [10,11,12,13,14,15,16,17,44].	Complex alleles remain vulnerable to genotype error, LD confounding and uncertain molecular function.
Segregation, endogenous perturbation and convergent natural evidence	Can connect an allele, molecular consequence and phenotype through independent observations [36,50,51].	Portability across breeds, pleiotropy and environment-dependent effects may remain unresolved.
Mendelian randomization (MR)	Tests whether genetically predicted variation in a molecular exposure, such as gene expression, is consistent with a directional causal effect on the complex trait [42,43,52,53,54].	Depends on instrument relevance, independence and exclusion-restriction assumptions; LD, horizontal pleiotropy, multiple local signals and weak instruments can mimic or distort mediation. MR therefore prioritizes causal hypotheses but does not by itself establish molecular mediation.

**Table 2:** Proposed Cattle Locus Dossier Standard: minimum information for reporting and reanalysis of cattle GWAS loci.

Reporting Domain	Minimum Information to Report
Phenotype	Trait definition, measurement method, biological unit, time window or physiological state, and relevant covariates.
Population	Breed or ancestry composition, sample size, sex where relevant, family structure and population used for discovery or validation.
Genome coordinates	Reference genome assembly, chromosome/contig, coordinate system and reference/alternative allele orientation.
Variant catalogue	Variant classes interrogated, including SNPs, indels and structural/non-reference variants where available.
Genotype quality	Genotyping or sequencing method, imputation reference panel, variant-level quality metrics and allele frequency by population.
Local association	Lead association, effect estimate and direction, conditional signals and definition of the analyzed locus.
Fine-mapping	Method, assumed number of causal signals, credible-set definition, posterior probabilities and priors where applicable.
Local LD	Cohort-specific LD matrix or equivalent local LD information, with the population and samples used to estimate it stated explicitly.
Haplotype and phasing	Phase of candidate alleles and the focal trait-associated haplotype; informative recombinant or family segregation data where available.
Molecular QTL	Molecular phenotype, tissue/cell type and physiological state, effect allele and direction, and evidence that the molecular-QTL signal is phased with the trait-associated haplotype.
Colocalization/MR	Method, model assumptions, handling of multiple signals and relevant sensitivity analyses.
Chromatin/cell-state evidence	Regulatory element, assay, tissue/cell population and biological state in which activity was observed.
Functional evidence	ASE, reporter assay, endogenous perturbation, editing, segregation or other allele-dependent testing, including experimental system and effect direction.
Structural variants	Variant type, breakpoint confidence, inserted sequence or copy-number state, graph path where applicable, and orthogonal validation method.
Competing explanations	Correlated candidate variants, alternative effector genes, possible pleiotropy or allelic heterogeneity, and untested variant classes.
Negative evidence	Relevant null or discordant findings, including tissues, populations or assays in which the proposed effect was not detected.
Current interpretation	Strongest claim justified by the evidence: associated locus, mechanistic candidate, functionally supported variant or experimentally supported causal variant.

**Table 3:** Representative examples of resolved and unresolved cattle locus interpretation.

Locus or Study	Main Contribution	Current Interpretation and Remaining Uncertainty
*DGAT1* K232A	Dense mapping, association and altered enzyme activity consistent with milk-composition effects [50].	Experimentally supported causal coding variant; portability and pleiotropic consequences remain trait- and population-dependent.
*ABCG2* Y581S	Fine localization, strong association and a biologically plausible transporter mechanism [51].	Strongly supported coding candidate; allele-level functional support is less extensive than for *DGAT1*.
*GHR*, *MSTN*, *PLAG1* and *PRL/PRLR*	QTL dissection, distinctive phenotypes, segregation and functional or physiological follow-up [66,67,70,71,72].	Benchmark loci with unusually informative alleles or pedigrees; the regulatory nucleotide remains less certain at *PLAG1*.
*MGST1* milk-fat locus	Sequence association plus mammary eQTL, with 17 correlated non-coding candidates [65].	Effector gene strongly supported; causal regulatory nucleotide remains unresolved.
Male-fertility molecular QTL	Sequence variants linked to expression and splicing in testis, epididymis and vas deferens [37].	Tissue-resolved mechanistic candidates; allele-level and phenotype-level validation remain limited.
Splice-disrupting variants	GWAS, computational prediction and massively parallel reporter testing [36].	Experimentally supported splice effects; organism-level causal interpretation is strongest for a subset.
Pangenome and long-read SV studies	Assembly discovery, graph genotyping, molecular QTL or large-cohort trait mapping [10,14,15,16,17,44].	Structural candidates expand the locus model; molecular and phenotype-level mechanisms remain locus-specific.
*L-LCORL* growth variants	Cross-species recurrence plus reported functional and mechanistic validation of truncating variants [73].	Allele-level function is strengthened; cattle breed portability, pleiotropy and organism-level trade-offs remain to be resolved.

## Data Availability

No new data were created or analyzed in this study. Data sharing is not applicable to this article.

## Abbreviations

The following abbreviations are used in this manuscript:

**Abbreviation**	**Definition**
ASE	Allele-specific expression
eQTL	Expression quantitative trait locus
FAANG	Functional Annotation of Animal Genomes
GWAS	Genome-wide association study
LD	Linkage disequilibrium
QTL	Quantitative trait locus
sQTL	Splicing quantitative trait locus
SV	Structural variant
